# Recent trends from the results of clinical trials on gastric cancer surgery

**DOI:** 10.1186/s40880-019-0360-1

**Published:** 2019-03-27

**Authors:** Takashi Kiyokawa, Takeo Fukagawa

**Affiliations:** Department of Surgery, Teikyo University School of Medicine, 2-11-1 Kaga, Itabashi-Ku, Tokyo, 173-8606 Japan

**Keywords:** Gastric cancer, Japanese Gastric Cancer Association, Randomized clinical trials, Para-aortic lymph nodes, D2 lymphadenectomy, D3 lymphadenectomy, Left thoraco-abdominal approach, Hiatal approach, Splenectomy, Bursectomy

## Abstract

The Japan Clinical Oncology Group has recently conducted large scale clinical trials with findings that have revealed pivotal strategies for the treatment of resectable gastric cancer surgery. These findings include the fact that D3 lymphadenectomy does not improve survival rates when compared to D2 lymphadenectomy, and it is not recommended for resectable gastric cancer. Also, a transhiatal approach is recommended, instead of the left thoraco-abdominal approach, for the treatment of adenocarcinoma of the esophago-gastric junction or gastric cardia which has invaded ≤ 3 cm of the esophagus. Gastrectomy with splenectomy and bursectomy had been recommended as a part of the D2 lymphadenectomy. However, the results of the recent clinical trials revealed that splenectomy should be avoided in total gastrectomy with D2 lymphadenectomy for proximal gastric cancer and that bursectomy should be avoided in gastrectomy with D2 lymphadenectomy for resectable gastric cancer. Both splenectomy and bursectomy were found to be unable to improve survival, but instead increased operative morbidity. These trials revealed that the above-mentioned invasive and aggressive procedures did not provide sufficient survival benefits and that gastric cancer surgery may be trending from an “invasive to less invasive” and “aggressive to more conservative” approach.

## Introduction

Although there has been remarkable progress in the treatment of gastric cancer, including in minimally invasive surgeries and chemotherapeutics, occurring in the recent years, however, gastrectomy with lymph node dissection still remains the only curative method for patients with gastric cancer. The Japanese Gastric Cancer Association (JGCA), inaugurated in 1962, has proposed several therapeutic strategies for gastric cancer which have historically established surgical standards in this field based on numerous data and efforts of several surgeons over the years [1].

The treatment of gastric cancer in Japan is conducted according to the Japanese Guideline of Gastric Cancer and the Japanese Classification of Gastric Carcinoma. Current editions are the 5th Japanese Gastric Cancer Treatment Guidelines and the 15th Japanese Classification of Gastric Carcinoma [2, 3]. When the first edition of the Japanese Gastric Cancer Treatment Guideline was published in 2001 [4], there was insufficient solid evidence regarding the therapeutic strategies based on clinical trials and scientific studies, as the Japanese therapeutic strategies for gastric cancer were mainly based from the experience of surgeons and through clinical practice. Even the efficacy of D2 dissection had not been properly evaluated by large scale clinical trials in Japan. Subsequent to the publication in 2001, several clinical questions have been verified by clinical trials mainly conducted by the Japan Clinical Oncology Group (JCOG). Some of their findings from conducting large scale clinical trials are gradually contributing to more standardized therapeutic strategies for gastric cancer. In this review, we discuss the recent trends of the results from JCOG trials on gastric cancer surgery.

## Para-aortic lymph nodes dissection (D3) vs. standard D2 lymphadenectomy

D2 lymphadenectomy, rather than D1 lymphadenectomy, has become the standard for the extent of lymphadenectomy to be performed for resectable gastric cancer, not only in East Asia but also in Western countries. While D2 lymphadenectomy has been performed routinely for years in Japan, D1 lymphadenectomy has been the standard lymphadenectomy performed in Western countries due to higher morbidity and mortality, and lower survival rate following D2 lymphadenectomy as compared to D1 lymphadenectomy [5–7].

However, between August 1989 and July 1993, the Dutch Gastric Cancer Group conducted a phase III trial on 711 locally advanced gastric cancer patients to compare the efficacy of D1 against D2 lymph node dissection [6, 8]. Though the surgical mortality was significantly high in the D2 lymphadenectomy group (10%) when compared to that of D1 lymphadenectomy (4%), the 15-year follow-up results showed that the postoperative gastric cancer-related deaths were significantly lower after D2 lymphadenectomy as compared to D1 lymphadenectomy (37% vs. 48%, respectively; *P* = 0.01). In addition, D2 lymphadenectomy was found to be associated with a lower locoregional recurrence (12% vs. 22%, respectively) [9]. This study demonstrated the benefit of D2 lymphadenectomy for advanced gastric cancer and resulted in D2 lymphadenectomy, without pancreatosplenectomy, to be performed in Western countries.

**Fig. 1:**
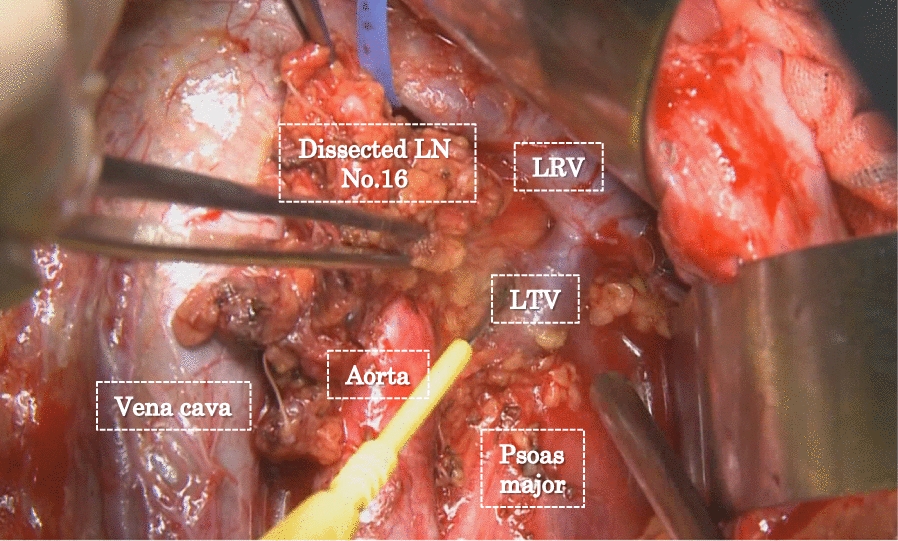
Para-aortic lymph node dissection. Illustration of the para-aortic lymph node dissection of the caudal part of the left renal vein during D2 lymphadenectomy. LRV, left renal vein; LN No. 16, lymph nodes at station 16; LTV, left testicular vein

However, it still remained unclear whether D2 lymphadenectomy plus para-aortic lymph node (PAN) dissection (D3) (Fig. 1) would improve the survival rate of surgically resected gastric cancer patients. There were some retrospective reports which have shown that patients with PAN metastasis had a long survival rate after D3 lymph node dissection [10–14]. For that reason, in the 1990s, Japanese surgeons in some institutions performed D3 lymph node dissection for almost all advanced gastric cancer patients with the “enthusiasm” or “passion” for improving the curative rate gastrectomies [13, 15]. The reasons for the surgeons to opt for D3 lymph node dissection was possibly because the mortality rate after D3 lymph node dissection in Japan was low, almost similar to that of D2 lymph node dissection, but with the advantage of retrieving more metastatic lymph nodes and expectation of increasing curative rate [13, 15, 16].

However, in 1995, a phase III open-label randomized clinical trial (RCT) performed by the JCOG, the JCOG 9501 study, for locally advanced gastric cancer revealed that routine D3 lymph node dissection did not improve the survival rate of patients with gastric cancer [16]. In that study, a total of 523 patients were assigned to compare the treatment of D2 versus D3 (D2 + PAN) lymph node dissection. The results showed that the surgical mortality rate was very low in both groups (0.8%). No significant difference was found between the two treatment groups in terms of 5-year recurrence-free survival (RFS) (62.6% vs. 61.7%, respectively; hazard ratio [HR] for recurrence in the group assigned to D3 was 1.08 and the 95% confidence interval [CI] was 0.83–1.42; *P* = 0.56), and overall survival (OS) [70.3% vs. 69.2%, respectively and the HR for death was 1.03 (95% CI 0.77–1.37; *P* = 0.85)], but the overall perioperative complication rate in the D3 group was higher than that in the D2 group (28.1% vs. 20.9%, respectively). Further, the observed minor surgery-related complications, such as ileus, lymphorrhea, left pleural effusion, were significantly higher in the D3 group (20.0% vs. 9.1%, *P* < 0.001).

Table 1 shows the results of other RCTs comparing D2 to D3 or D4 (D3+) lymph node dissection [16–19]. These trials showed that there were no significant survival rate benefit for performing PAN dissection in curable gastric cancer patients and simultaneously revealed its association to a higher surgical morbidity. As such, gastrectomy with D2 lymphadenectomy has been considered as the standard routine lymphadenectomy for locally advanced gastric cancer.

**Table 1:** The results of randomized clinical trials comparing the efficacy of D2 lymphadenectomy to D3 or D4 (D3+) lymphadenectomy

Author	Year	Number of patients	Country	Tumor depth	Comparative arm^a^	Survival results	Morbidity D2 vs. D3 (%)
Maeta et al. [17]	1997	70	Japan	T3–T4	D2+ vs. D4 (D3+)	NS	26.0 vs. 40.0
Wu et al. [33]	2006	221	Japan, Korea, China, Taiwan	T2–T4/N1–3	D2 vs. D4 (D3+)	Unknown	7.3 vs. 17.1
Kulig et al. [18]	2007	550	Poland	T1–T3	D2 vs. D3	NS	27.7 vs. 21.6
Sasako et al. [16]	2008	523	Japan	T2–T4	D2 vs. D3	NS	20.9 vs. 28.1
Yonemura et al. [19]	2008	269	Japan, Korea, Taiwan	T2–T4	D2 vs. D3	NS	Mortality: 0.7 vs. 3.7

T, depth of tumor infiltration; N, number of metastasized lymph nodes; D, types of lymphadenectomy; NS: not significant

^a^The different types of lymphadenectomies performed in the comparative arms of the respective randomized clinical trials: D2+: D2 lymphadenectomy plus dissection of lymph nodes located at the hepatoduodenal ligament, in the retro-pancreatic space and along the vessels of the transverse mesocolon. D3: D2 lymphadenectomy plus dissection of lymph nodes located at the para-aortic lymph node dissection from the upper margin of the celiac trunk to the lower margin of the left renal vein. D4 (D3+): D2 lymphadenectomy plus dissection of lymph nodes located at the para-aortic lymph nodes from the aortic hiatus to the aortic bifurcation (hepatoduodenal ligament, in the retro-pancreatic space and along the vessels of transverse mesocolon)

However, the effect of the D3 dissection on gastric cancer patients with PAN metastasis is still debatable. In 2004, the JCOG launched a phase II trial for patients with PAN metastases which had preoperative S-1/cisplatin chemotherapy followed by D3 dissection (JCOG 0405 study) [20]. The results of this study demonstrated a relatively high 5-year OS rate (53%), even though the proportion of pathological metastasis in the group 3 lymph nodes (extent of lymph node N3 in the Japanese Classification of Gastric Carcinoma—2nd English edition), including PAN, was 31% [21]. In another phase II trial, with a relatively short follow-up, Wang et al. [22] showed that the use of preoperative chemotherapy with capecitabine and oxaliplatin followed by D3 gastrectomy for advanced gastric cancer patients with PAN metastases had a 1-year OS rate of 67.9% and a median OS of 29.8 months [22]. Since the OS rate observed in these two reports were relatively high, this suggests that D3 lymphadenectomy with preoperative chemotherapy was beneficial for some patients with PAN metastasis but, more investigations are needed in regard to the proper patient selection.

## Left thoraco-abdominal vs. hiatal approach

**Fig. 2:**
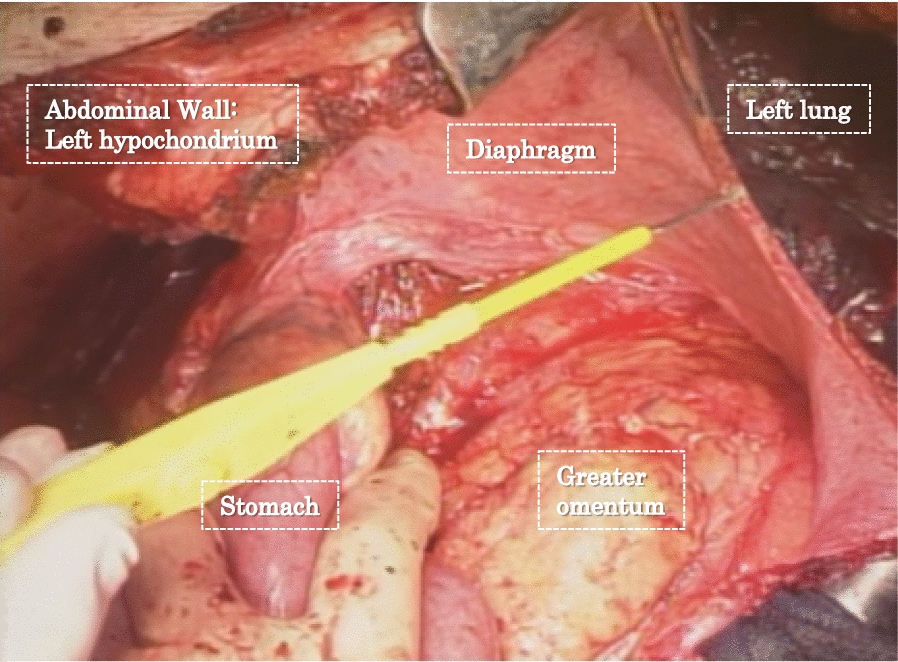
The left thoraco-abdominal approach. Illustration of the transection of the diaphragm for adenocarcinoma at/near the esophagogastric junction

The surgical approach for the treatment of cancer of the esophagogastric junction (EGJ) was controversial in the 1990s. Some institutions preferred the left thoraco-abdominal (LTA) approach (Fig. 2) to the transhiatal (TH) approach to perform lymph node dissection in the lower mediastinal field and for obtaining a safer surgical margin. Others preferred the TH approach considering that it had a lower postoperative morbidity and that the prognosis for patients with metastasis in the lower mediastinum was poor.

In the year 1995 in Japan, a phase III open-label RCT (the JCOG9502 study) was initiated to compare the TH and LTA approaches for patients with EGJ cancer invading the esophagus by 3 cm or less [23, 24]. The trial randomly assigned 82 and 85 patients to the TH and LTA group, respectively. However, the trial was stopped after the first interim analysis because patients assigned to the LTA group were unlikely to have an improved OS compared with those assigned to the TH group, and that the LTA group had a higher morbidity rate (LTA group, 49% vs. TH group, 34%; *P *= 0.060) [24]. For a selected of six major complications (pancreatic fistula, abdominal abscess, pneumonia, anastomotic leak, pulmonary emphysema, and mediastinitis), the incidence was significantly higher following the LTA approach as compared to the TH approach (41% in the LTA group vs. 22% in the TH group; *P* = 0.008) [24]. The 5-year disease-free survival (DFS) rate was higher in the TH group, but not significantly different from the LTA group [47% vs. 37%, respectively; the HR for the LTA group compared with the TH group was 1.28 (95% CI 0.87 to 1.89; *P* = 0.215)]. Also, the 5-year OS was higher in the TH group, but without any observed significant statistical difference [51% in the TH group vs. 37% in the LTA group; the HR for the LTA compared with the TH approach was 1.42 (95% CI 0.98–2.05; *P* = 0.060)].

These results showed that the LTA technique could not significantly improve the OS or DFS rate when compared with the TH approach and were associated with greater morbidity and mortality. Therefore, based on the finding of the JCOG9502 study, the LTA approach is not recommended in the treatment of adenocarcinoma of the EGJ or gastric cardia with esophageal invasion of ≤ 3 cm.

## Splenectomy in total gastrectomy for proximal gastric carcinoma

Splenectomy aiming for complete lymphadenectomy at the splenic hilum, used to be performed in Japan, because the complete lymph node dissection was not technically feasible without a splenectomy. However, its overall survival benefit was unclear, although splenectomy in total gastrectomy was associated with an increase in operative morbidity and mortality.

Cuschieri et al. [25] reported the results of an RCT—comparing the postoperative morbidity and mortality after D1 and D2 lymphadenectomy—consisting of 400 patients with curable gastric cancer. They found that performing splenectomy led to a high surgical morbidity (54%) and mortality (16%). Multivariate analyses showed that conducting splenectomy was the possible cause of a lower survival rate, in contrast to when a splenectomy was not performed. However, the difference was not statistically significant, for which the HR of the splenectomy group compared with the preservation group was 1.36 (95% CI 0.97–1.90; *P* = 0.07). This result thereby suggested that splenectomy may not contribute to a survival benefit.

However, despite the mortality was relatively high after splenectomy, there were some RCTs which showed that patients who had undergone splenectomy had a better survival than those having spleen-preserving gastrectomies if the perioperative splenectomy mortality was low, although the obtained results were not statistically significant (Table 2).

**Table 2:** Results of the randomized clinical trials comparing the efficacy of gastrectomy with and without splenectomy

Author	Year	Number of patients	Country	Morbidity		Mortality		Survival difference
				Splenectomy (%)	Non-splenectomy (%)	Splenectomy (%)	Non-splenectomy (%)	
Cuschieri et al. [7, 25]	1999	400	UK	54.0^a^	28.0^a^	16.0^a^	4.0^a^	NS
Csendes et al. [34]	2002	187	Chile	50.0^a^	39.0^a^	4.4	3.1	NS
Yu et al. [26]	2006	207	Korea	15.4	8.7	1.9	1.0	NS
Sano et al. [29]	2017	505	Japan	30.3^a^	16.7^a^	0.4	0.8	NS

NS, not significant

^a^Demonstrated statistical significance between the respective comparative groups

Further, Csendes et al. [22] reported the results of an RCT which compared D2 total gastrectomy versus D2 total gastrectomy plus splenectomy in 187 gastric cancer cases. They found a slightly better 5-year survival rate regarding the splenectomy group as compared to those with spleen preservation (42% vs. 36%, respectively), with an observed operative mortality similar in both groups (4.4% vs. 3.1%, respectively). Yu et al. [26] reported the results of a RCT for splenectomy versus splenic preservation in 207 patients with proximal gastric cancer and found a slightly better survival rate with for those with splenectomy (54.8% vs. 48.8%, respectively; *P *= 0.503), with low surgical mortality (1.9% vs. 1.0%, respectively).

In Japan, splenectomy was routinely performed with total gastrectomy for years as the observed mortality was very low but, until there were some retrospective studies which showed that splenectomy did not contribute to survival benefit [27, 28]. Finally, in 2002, a phase III open-label RCT, the JCOG0110 study, was initiated on 505 patients to compare their survival after total gastrectomy with and without splenectomy for proximal gastric cancer [29]. Their recent published results showed that splenectomy was associated with a high morbidity (30.3%) but low mortality (0.4%) and did not improve survival rates. The 5-year OS rates were 75.1% and 76.4% in the splenectomy and spleen preservation groups, respectively. The HR for death in the spleen preservation group, when compared to the splenectomy group, was 0.88 (90.7% CI 0.67–1.16, 1-sided *P* for non-inferiority = 0.025). The 5-year RFS of the splenectomy and spleen preservation groups were 68.4% (95% CI 62.3–73.7) and 70.5% (95% CI 64.4–75.7), respectively, and the HR for the spleen preservation to splenectomy group was 0.87 (95% CI 0.65–1.17). Based on these results, splenectomy is not recommended in total gastrectomy since it has been found to increase operative morbidity without significantly improving survival.

## Bursectomy for advanced gastric cancer

**Fig. 3:**
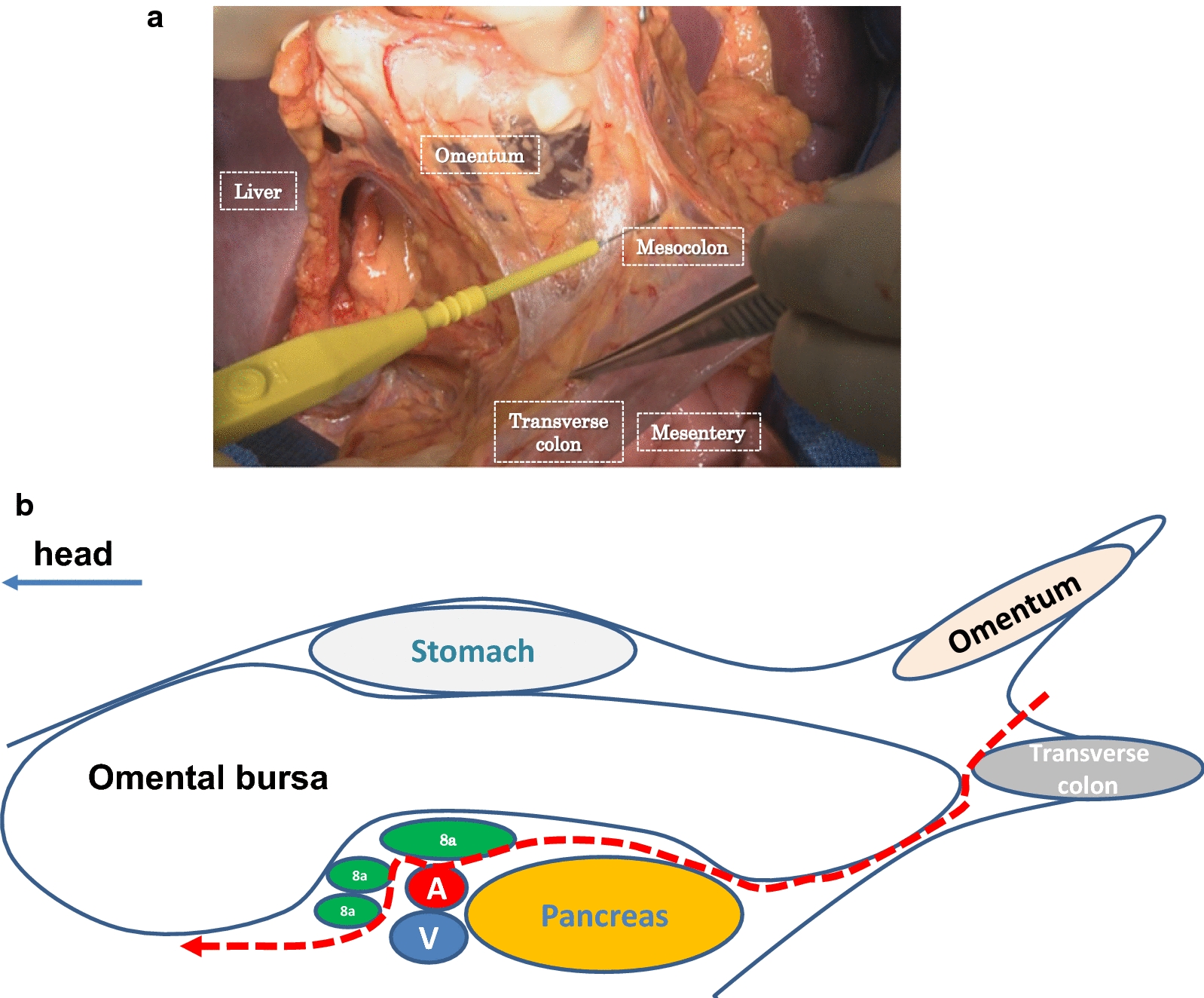
Illustrations of bursectomy for advanced gastric cancer. **a** Dissecting the anterior layer of the transverse mesocolon. **b** Schema of the bursectomy. The red arrow represents the dissection line for bursectomy. 8a, lymph nodes at station 8a: A, common hepatic artery; V, splenic vein

The role of performing bursectomy, in which the peritoneal lining covering the pancreas and the anterior plane of the transverse mesocolon is dissected (Fig. 3a, b), for the prevention of peritoneal metastasis in gastric cancer has long been controversial. A retrospective study of 254 patients in Japan showed that gastrectomy with bursectomy had a better 5-year OS than that of without bursectomy, although the difference was not statistically significant [85.8% vs. 80.8%, respectively (HR 0.82; 95% CI 0.37–1.74; *P* = 0.60)] [30].

To demonstrate the survival non-inferiority of gastrectomy with the omission of bursectomy, an RCT was initiated in 2002 by the Osaka University Clinical Research Group for Gastroenterological Surgery, which enrolled two hundred and ten patients with cT2–T3 gastric adenocarcinoma [31]. The obtained 3-year OS rates were 85.6% in the bursectomy group and 79.6% in the non-bursectomy group. The HR for death without bursectomy was 1.44 (95% CI 0.79–2.61; *P* = 0.443 for non-inferiority). Among 48 pathologically serosa-positive (pT3–T4) patients, the 3-year OS was 69.8% for the bursectomy group and 50.2% for the non-bursectomy group, conferring to an HR for death of 2.16 (95% CI 0.89–5.22; *P* = 0.791 for non-inferiority). A greater number of patients in the non-bursectomy group were found to have peritoneal recurrences as compared to the bursectomy group (13.2% vs. 8.7%). These results suggested that the bursectomy group tended to have a better OS than that of the non-bursectomy group, although they were not statistically significant.

A large-scale phase III open-label RCT, the JCOG1001 study, was conducted between June, 2010 and March, 2015, to compare the survival benefit of gastrectomy with and without bursectomy and to clarify this issue [32]. A total of 1204 cT3–T4a gastric cancer patients were enrolled but no survival differences between the two groups were found. The 5-year OS was 76.7% (95% CI 72.0–80.6) in the non-bursectomy group and 76.9% (95% CI 72.6–80.7) in the bursectomy group. The HR for death in the bursectomy group as compared to the non-bursectomy group was 1.05 (95% CI 0.81–1.37; one-sided *P* = 0.65). The 5-year RFS was 69.3% (95% CI 64.8–73.3) in the non-bursectomy group and 68.0% (95% CI 63.5–72.1) in the bursectomy group. The HR for recurrence in the bursectomy group versus the non-bursectomy group was 1.07 (95% CI 0.86–1.33, two-sided *P* = 0.54). Contrary to the above-mentioned results of the Osaka University Clinical Research Group, this large-scale trial could not demonstrate a survival benefit of bursectomy over the non-bursectomy group in the treatment of resectable gastric cancer. A possible reason for the difference in the results shown between these two RCTs may be the advent of postoperative chemotherapy. In the JCOG1001 study, the patients were allowed to receive adjuvant S-1 chemotherapy, but no adjuvant treatments were allowed in the Osaka Trial [32]. Since postoperative chemotherapy is performed for almost all advanced gastric cancer patients nowadays, bursectomy may not be recommended for resectable advanced gastric cancer.

## Discussion

Since the racial background of Japanese patients has some advantages as compared to Western countries’ patients in terms of low BMI and lesser co-morbidities, aggressive and meticulous gastric cancer surgeries had been easily performed in Japan. In addition, the high incidence of gastric cancer in Japan has contributed to a big accumulation of surgeons’ experience in surgical oncology and techniques of performing the operations. The development of gastric cancer surgery in Japan had been based on those reasons. Although the Japanese-style gastrectomy might be difficult for Western surgeons to perform on their Western patients for the above-mentioned reason, recent standardization of gastric cancer surgery based on Japanese clinical trials could still be applied in other countries and possibly with the same oncological benefit and low post-operative mortality with certain extent of accumulation of the surgeons’ experience.

Based on the results of these four clinical trials—the JCOG9501 (D2 vs. D2 + PAN dissection), JCOG9502 (THA vs. LTA), JCOG0110 (without splenectomy vs. with splenectomy), and JCOG1001 (bursectomy vs. non-bursectomy— the D2 lymphadenectomy, THA approach, spleen-preservation gastrectomy and non-bursectomy (omentectomy) are recommended as standard strategies for resectable gastric cancer surgery in each of their respective application. It is interesting to note that less invasive methods were adopted among the comparative arms in each of these major clinical trials. Japanese surgeons have performed D2 + PAN dissection, the THA approach, splenectomy, and bursectomy aiming for oncological benefit over many years; however, such invasive and aggressive procedures have now been proven to be without survival benefits and surgeons are now gradually accepting these scientific-based results.

Less invasive and conservative procedures may be the forth-coming recent trend in gastric cancer surgery. However, these results of the clinical trials did not imply the advantage of using laparoscopy or robot for minimally invasive surgeries (laparoscopic and robotic surgeries). The results did not mean that the less surgical stress contributed to the better results. Discussion regarding less invasive surgeries should not include the debate on minimally invasive surgeries (laparoscopic and robotic surgeries) for gastric cancer. Therapeutic strategies for oncological treatment should be the same across all surgical modalities.

Based on the results of the recent clinical trials discussed in this review, we can observe that the treatment strategies in gastric cancer surgery are trending from an “invasive to a less invasive” and “aggressive to a more conservative” approach.
